# A New Strategy for Multitarget Drug Discovery/Repositioning Through the Identification of Similar 3D Amino Acid Patterns Among Proteins Structures: The Case of Tafluprost and its Effects on Cardiac Ion Channels

**DOI:** 10.3389/fphar.2022.855792

**Published:** 2022-03-18

**Authors:** Alejandro Valdés-Jiménez, Daniel Jiménez-González, Aytug K. Kiper, Susanne Rinné, Niels Decher, Wendy González, Miguel Reyes-Parada, Gabriel Núñez-Vivanco

**Affiliations:** ^1^ Center for Bioinformatics, Simulations and Modelling, Faculty of Engineering, University of Talca, Talca, Chile; ^2^ Computer Architecture Department, Universitat Politécnica de Catalunya, Barcelona, Spain; ^3^ Barcelona Supercomputing Center, Barcelona, Spain; ^4^ Institute for Physiology and Pathophysiology, Philipps-University Marburg, Marburg, Germany; ^5^ Millennium Nucleus of Ion Channels-Associated Diseases (MiNICAD), Universidad de Talca, Talca, Chile; ^6^ Centro de Investigación Biomédica y Aplicada (CIBAP), Escuela de Medicina, Facultad de Ciencias Médicas, Universidad de Santiago de Chile, Santiago, Chile; ^7^ Facultad de Ciencias de la Salud, Universidad Autónoma de Chile, Talca, Chile; ^8^ Departamento de Ciencias Naturales y Tecnología, Universidad de Aysén, Coyhaique, Chile

**Keywords:** polypharmacology, binding site similarity, cardiac ion channels, tafluprost, binding site comparisons

## Abstract

The identification of similar three-dimensional (3D) amino acid patterns among different proteins might be helpful to explain the polypharmacological profile of many currently used drugs. Also, it would be a reasonable first step for the design of novel multitarget compounds. Most of the current computational tools employed for this aim are limited to the comparisons among known binding sites, and do not consider several additional important 3D patterns such as allosteric sites or other conserved motifs. In the present work, we introduce Geomfinder2.0, which is a new and improved version of our previously described algorithm for the deep exploration and discovery of similar and druggable 3D patterns. As compared with the original version, substantial improvements that have been incorporated to our software allow: (i) to compare quaternary structures, (ii) to deal with a list of pairs of structures, (iii) to know how druggable is the zone where similar 3D patterns are detected and (iv) to significantly reduce the execution time. Thus, the new algorithm achieves up to 353x speedup as compared to the previous sequential version, allowing the exploration of a significant number of quaternary structures in a reasonable time. In order to illustrate the potential of the updated Geomfinder version, we show a case of use in which similar 3D patterns were detected in the cardiac ions channels NaV1.5 and TASK-1. These channels are quite different in terms of structure, sequence and function and both have been regarded as important targets for drugs aimed at treating atrial fibrillation. Finally, we describe the *in vitro* effects of tafluprost (a drug currently used to treat glaucoma, which was identified as a novel putative ligand of NaV1.5 and TASK-1) upon both ion channels’ activity and discuss its possible repositioning as a novel antiarrhythmic drug.

## Introduction

Although most novel drugs are still developed using the “magic bullet” paradigm, which involves highly selective profiles, chemical compounds are naturally promiscuous in practice. Indeed, most therapeutically beneficial agents interact with more than one molecular target (Feldmann and Bajorath, 2020). Interestingly, this promiscuity is now, in some cases, considered an advantageous feature and is proactively pursued. Thus, many drug development initiatives are focused on the design of multitarget compounds with a polypharmacological profile that may improve drug-based treatments’ efficacy and/or safety (Ramsay et al., 2018; Proschak et al., 2019). Unfortunately, the design of drugs with multiple activities on a selected handful of different protein structures remains a significant experimental and computational challenge (Konc, 2019). Recent reports have proposed several strategies to identify multitarget drugs, such as clinical observations and target combinations based on phenotypic screening. Bioinformatics is also a useful tool to address these challenges through molecular modelling techniques for detecting similar targets, machine learning methods to find disease-related targets, target-fishing using molecular docking, ligand-based pharmacophore searching, virtual screening simulations, and the search of binding sites similarities (Ma et al., 2010; Ren et al., 2021; Stępnicki et al., 2021). Finding compounds with multitarget action on related proteins which share a similar function, folding or binding sites, is currently an accessible task. Unfortunately, complex diseases often comprise a wide range of evolutionary distant and structurally different proteins where current methods are not entirely precise. For example, neuropsychiatric, cardiac or autoimmune disorders (among others), including atrial fibrillation or major depression, are complex diseases that often encompass dysfunctions in a wide range of types of proteins such as ion channels, enzymes, transporters, globular proteins, etc. (Bolognesi, 2019; Konc, 2019). Thus, the identification of conserved/similar sites (or more broadly three-dimensional (3D) patterns, defined as a local structural arrangement of amino acids) among a set of proteins (related or not between them), can be useful for the rational design/repositioning of polypharmacological drugs (Konc, 2019; Adasme et al., 2020; Li et al., 2021). In this context, tools such as G-LoSA (Lee and Im, 2016), Geomfinder (Núñez-Vivanco et al., 2016), 3D-PP (Valdés-Jiménez et al., 2019) and others (Ehrt et al., 2016; Ehrt et al., 2018), which work regardless of information about known ligands, binding sites, sequence similarity, or structural folding of the proteins, improve the chances of finding similar 3D patterns among very different or unrelated targets. Although some of these similarities may appear by chance, others might represent distant evolutionary relationships and correspond, for instance, to secondary binding sites. These sites have recently gained attention for the rational design of polypharmacological allosteric modulators (Abdel-Magid, 2015; Meysman et al., 2015; Reyes-Parada and Iturriaga-Vasquez, 2016; Wakefield at al., 2019). Indeed, by using our in-house algorithm Geomfinder (Núñez-Vivanco et al., 2016), we have reported the finding of some similar 3D patterns among very different protein structures, which cannot be observed through other structural tools or with sequence-based methods. Noteworthy, in the original version of Geomfinder, the residues of each detected 3D pattern could only be part of one specific protein chain. However, it is well known that several important 3D patterns (e.g. binding sites, catalytic sites) are located either at the interface between different subunits of a single target (Lee et al., 2017) or at the oligomer interface in multimeric proteins (Baek et al., 2017).

In the present work, we introduce Geomfinder2.0, which is a new and improved version of our previous tool for the deep exploration and discovery of similar and druggable 3D patterns. Thus, the possibility of exploring 3D patterns formed by different chains of a quaternary protein structure is one of the major novel features of this version. Also, the updated algorithm includes a function that predicts the zone where similar 3D patterns may be druggable (Le Guilloux et al., 2009). Thus, a 3D pattern with a high level of druggability found in several protein structures might be used as the input for the design of multitarget compounds using approaches such as Pocket-Based Drug Design (Zheng et al., 2013). From a computational perspective, the currently available version of Geomfinder has been fully migrated from Python 2.7 to C++ language and parallelized using the shared memory programming model OpenMP (Dagum and Menon, 1998). These improvements allowed a speedup of up to 353x. Furthermore, as a functional enhancement, Geomfinder can now compute several pairs of comparisons simultaneously.

In addition, in order to illustrate the potential of the updated Geomfinder version, we show a case of use in which similar 3D patterns were detected in two cardiac ion channels, specifically NaV1.5 and TASK-1, which are selective for sodium or potassium, respectively. These channels have been regarded as important targets for drugs aimed at treating atrial fibrillation (Sossalla et al., 2010; Wiedmann et al., 2021). Finally, we describe the *in vitro* effects of tafluprost (a putative ligand identified after a receptor-based drug search approach) upon both ion channels’ activity and discuss its possible repositioning as a novel antiarrhythmic drug.

## Materials and Methods

### Computational Methods

#### Software Improvements

**FIGURE 1:**
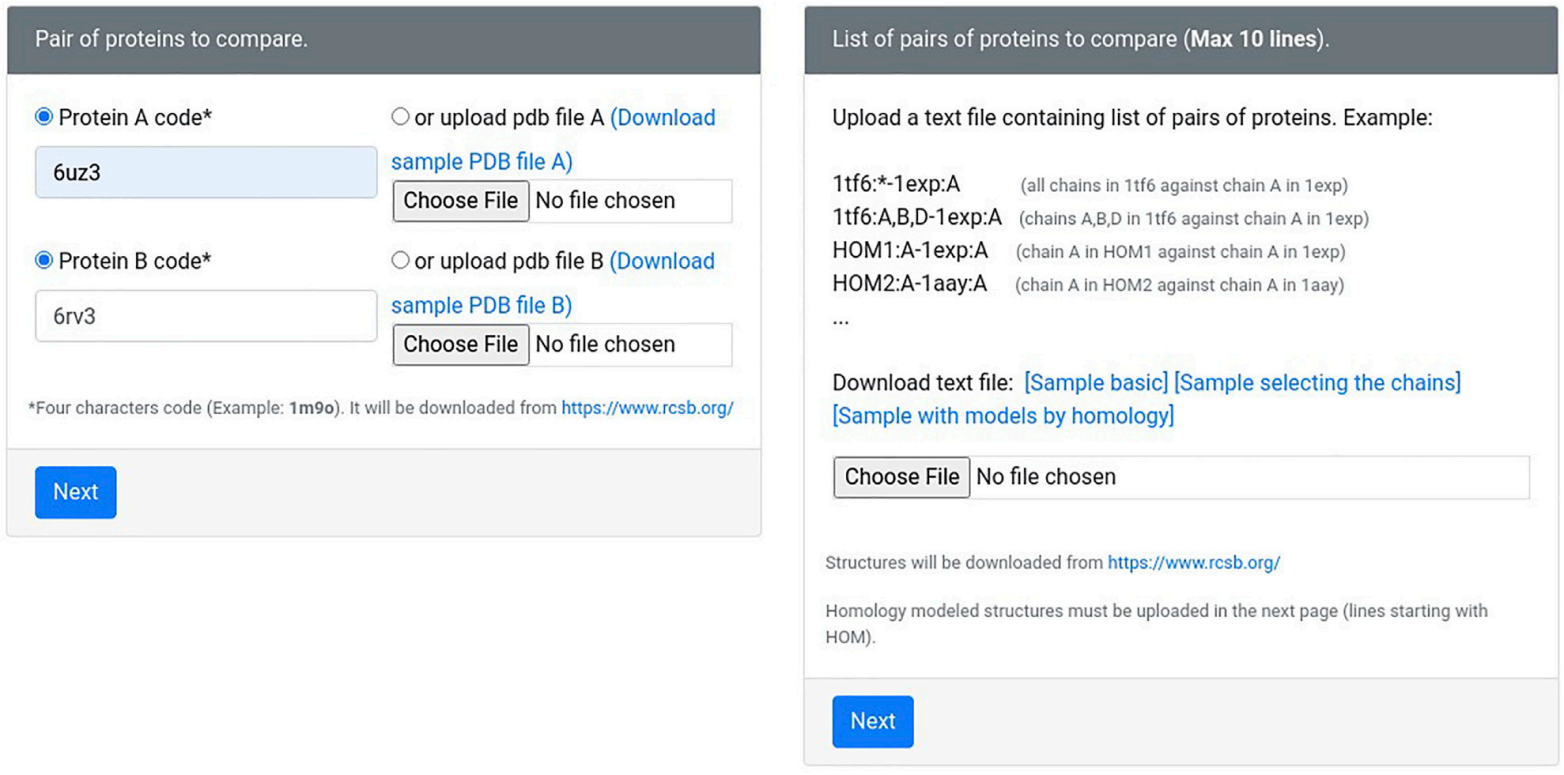
The user may request the measures between two protein structures or within a list of pairs of proteins. Also, the protein structures can be both experimentally solved (downloaded from Protein Data Bank) or homology models (uploaded by the user).

**FIGURE 2:**
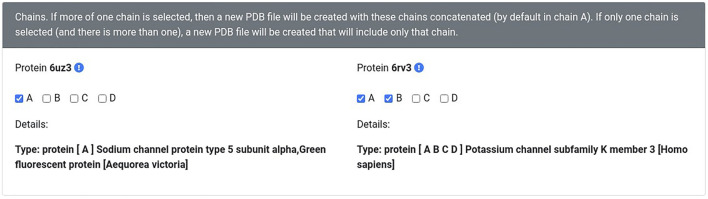
The user can concatenate different protein chains. The information concerning chains, names, and the source organism of each protein is obtained from the Protein Data Bank or from the same PDB file (in the case of homology models).

**FIGURE 3:**
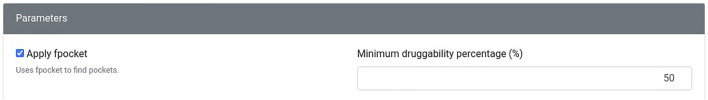
The user can select the application of Fpocket on the exploration of pockets. In addition, if this option is activated, a minimum druggability percentage must be included.

The new version of Geomfinder includes all geometrical characteristics and processes described previously (Núñez-Vivanco et al., 2016) and incorporates new features and substantial improvements. In the Supplementary Figure S1, the implemented architecture and essential components and services of Geomfinder are shown. Remarkably, the input can be a list of pairs of structures (always as PDB files) in this new version. This new feature makes it possible to find similar 3D patterns simultaneously in several pairs of protein structures submitted by the user (Figure 1). Another essential feature of the new version is the possibility of searching for 3D patterns in the interface between two or more protein chains/subunits. To this end, in the submission process, the user can select one particular chain of each or concatenate either all or some of the structure’s chains (Figure 2). Also, it is now possible to search 3D patterns only in those zones of the proteins where the cavities detected achieve the user-defined druggability score threshold, which is interpreted as the probability of the cavity to bind a drug and alter its normal activity (Pérot et al., 2010; Schmidtke and Barril, 2010). This preprocessing step is an optional parameter (Figure 3) calculated by the Fpocket algorithm (Le Guilloux et al., 2009), considering features such as the size, the hydrophobicity, and the normalized polarity of the residues lining the cavity. Technically, several sections of the algorithm were also optimized. The original version of Geomfinder (PythonThreading) was developed in Python 2.7 using threads as a parallel strategy. Searching for a better performance, three new versions were built: PythonMultiprocessing using Python 2.7 with multiprocessing, C++/Pthreads using C++ and POSIX threads, and C++/OpenMP using C++ with OpenMP annotations. These versions were analyzed (using a 32-CPU machine with hyperthreading activated) against a sequential version of Geomfinder implemented with benchmarking purposes.

#### Detection of Similar 3D Amino Acid Patterns Using Geomfinder

Searching for similar 3D amino acid patterns begins with the creation of a virtual grid of coordinates on each protein structure. Then, using all geometrical centers of all side chains of the residues, Geomfinder performs a residues grouping step depending on the distance between each virtual coordinate. With this, hundreds of 3D amino acid patterns are defined in each protein structure. After that, for each 3D pattern, four descriptors are measured: a) The list of distances between the geometric centers of the side chains of all the residues forming the 3D pattern; b) the sum of the short and medium-range of non-bonded energy of each residue forming the 3D pattern; c) the list of the residues forming a 3D pattern; and d) the list of the distances constituting the shortest pathway necessary to go over all the residues lining the 3D pattern. Finally, all pairs of 3D patterns identified in the two tested proteins are compared using an all-versus-all approach. Thus, at the end of the analysis, each pair of the 3D pattern has a final similarity score named GScore. The GScore is defined as a combination of the similarities (S) of the four descriptors:

$$GScore=SDist*D_{p}+\;SNbE*C_{p}+\;STsp*T_{p}+\;SSc*S_{p}$$

which is calculated as the relative changes on each pair of the 3D pattern as follows:

$$SDist=\;\frac{\left|Dist_{A}\;\cap Dist_{B}\right|}{\max\left(\left|Dist_{A}\right|,\left|Dist_{B}\right|\right)}\;\;\;\;\;\;\;\;\;\;SNbE=\;\frac{\min\left(\left|NbE_{A}\right|,\left|NbE_{B}\right|\right)}{\max\left(\left|NbE_{A}\right|,\left|NbE_{B}\right|\right)}$$

$$STsp=\;\frac{\left|Tsp_{A}\cap Tsp_{B}\right|}{\max\left(\left|Tsp_{A}\right|,\left|Tsp_{B}\right|\right)}\;\;\;\;\;\;\;\;SSc=\;\frac{\left|Sc_{A}\cap Sc_{B}\right|}{\max\left(\left|Sc_{A}\right|,\left|Sc_{B}\right|\right)}$$

SDist, SNbE, STsp, and SSc, are the partial scores of similarity of the distances, the non-bonded energies, the perimeter, and the sequence components, between any two 3D patterns.

#### Receptophore Determination

As we have previously proposed (Núñez-Vivanco et al., 2018), a “receptophore” can be defined -by analogy with the pharmacophore concept-as a 3D ensemble (present in two or more receptors), of molecular, steric, and electronic features that ensure the optimal molecular interactions with a common promiscuous ligand. Therefore, here we describe how we determine it from the local similarities identified with Geomfinder. The method consists basically of the structural alignment of the similar 3D patterns identified between the protein structures. This process was performed using the external computational methods PocketAlign and MultiBind (Shulman-Peleg et al., 2008; Yeturu and Chandra, 2011). This approach finds the best match of physicochemical properties among the residues forming each site. PocketAlign carries out multiple alignments between the similar 3D patterns detected to recognize similar amino acids matches. Then, numerous structural rearrangements of superimposed binding sites are applied to find the best structural fit. Briefly, this method consists of two main processes: a) the preprocessing of the features of each 3D pattern and hashing them into a table; and b) the recognition of the similar features in the objects of the hash table. In the preprocessing, each amino acid is denoted by pseudocenters (X, Y, and Z coordinates), which provides a unique physicochemical property to the binding site: hydrogen-bond donor, hydrogen-bond acceptor, mixed donor/acceptor, hydrophobic aliphatic or aromatic contacts. Finally, MultiBind performs a combination of multiple superimposed binding site conformations to find common patterns. Because 3D patterns that originate the receptophore are not necessarily identical, different residues might be partially aligned if the overall structural alignment score turns out to be maximized with that arrangement. After that, to generate the final receptophore, all equivalent amino acids between both 3D patterns (same physicochemical group: polar, non-polar, positively or negatively charged) appearing aligned or superimposed, are manually merged in the resultant PDB file. In contrast, all non-equivalent amino acids are preserved in the final receptophore.

#### Receptor/Pocket Based Virtual Screening

The previously defined receptophore was used to perform a virtual screening analysis through the computation tool e-LEA3D (Douguet, 2010). This program can create new molecules with a fragment-based approach or evaluate a user-defined data set of compounds. In our case, we used the “FDA-approved” data set to determine if some of these compounds exhibit affinity for the receptophore. Thus, each molecule’s fitness was evaluated via a function that inputs the molecular structure and returns a numeric score. The evaluation can integrate a selected number of molecular properties and/or a protein-ligand docking score calculated by the program PLANTS (Korb et al., 2009). After that, a list of FDA-approved drugs, ranked by their affinity for the receptophore, was obtained and analyzed for further experiments.

#### Protein Structures

For the case of use, we employed the crystallographic protein structures (obtained from the Protein Data Bank) of the sodium channel NaV1.5 (PDBid: 6UZ3, resolution 3.50 Å) and the two-pore domain potassium ion channel TASK-1 (PDBid: 6RV3, resolution 2.90 Å). Only chain A was selected in the case of NaV1.5, whereas for TASK-1, chains A and B were concatenated. All details of input parameters and results can be found at https://geomfinder2.appsbio.utalca.cl/result/11598984329232/

### Pharmacological Methods

#### Drugs and IC50 Values

Tafluprost was purchased from Merck. All other reagents used were of analytical grade. Tafluprost was dissolved in DMSO and added to the external solution just before the recordings. The IC50 (or EC50) were determined from Hill plots using up to five concentrations for each construct and are expressed as mean ± SD coming from the different replicates measurements (*n* = 3-9 replicates).

#### Oocyte Preparation and cRNA Injection

All procedures performed in this study involving animals were carried out in accordance with the EU Directive 2010/63/EU for animal experiments. The work with *Xenopus laevis* at the University of Marburg with all experimental protocols were approved by the Regierungspräsidium Gießen, Germany (V54-19c20 15 h 02 MR 20/28 Nr.A 4/2013).

Oocytes were obtained from anesthetized *Xenopus laevis* frogs and incubated in OR2 solution containing in mM: 82.5 NaCl, 2 KCl, 1 MgCl2, 5 HEPES (pH 7.5) substituted with 2 mg/ml collagenase II (Sigma) to remove residual connective tissue. Then the oocytes were stored at 18°C in ND96 supplemented with 50 mg/L gentamycin, 274 mg/L sodium pyruvate, and 88 mg/L theophylline.

Oocytes were each injected with either 50 nL of cRNA of human TASK-1 (KCNK3, NM_002246) or 10 ng of cRNA of human Nav1.5 (hH1, M77235), as previously described (Ortiz-Bonnin et al., 2016; Rinné et al., 2019).

#### Two-Electrode Voltage Clamp Recordings

Two-electrode voltage clamp recordings were performed at room temperature (20–22°C) with a TurboTEC 10CD (npi) amplifier and a Digidata1200 Series (Axon Instruments) as analog/digital converter, as previously described (Ortiz-Bonnin et al., 2016; Rinné et al., 2019). Briefly, micropipettes were made from borosilicate glass capillaries GB150TF-8P (Science Products) and pulled with a DMZ Universal Puller (Zeitz). Recording pipettes had a resistance of 0.5–1.5 MΩ when filled with 3M KCl solution.

For both, TASK-1 or NaV1.5 channel measurements, recording solution ND96 contained in mM: 96 NaCl, 2 KCl, 1.8CaCl2, 1 MgCl2, 5 HEPES (pH 7.5). In the case of TASK-1 channel, block was analyzed with voltage steps from a holding potential of −80 mV. A first test pulse to 0 mV of 1 s duration was followed by a repolarizing step to −80 mV for 1 s directly followed by another 1 s test pulse to +40 mV. The sweep time interval was 10 s. For NaV1.5 channel, block was analyzed with voltage steps from a holding potential of −120 mV. A first depolarizing pulse to −10 mV of 20 ms duration, then, after holding at −40 mV for 4s, a 20 ms step at −120 mV was carried out. The sweep time interval was 10 s.

Tafluprost at different concentrations was evaluated on channel currents. Stability in recordings was monitored prior to the addition of compounds, which were removed from the bath to show recovery.

Data were acquired with Clampex 10 (Molecular Devices) and analyzed with Clampfit 10 (MolecularDevices) and Origin 7 (OriginLab Corp.).

## Results

### Software Optimization

**FIGURE 4:**
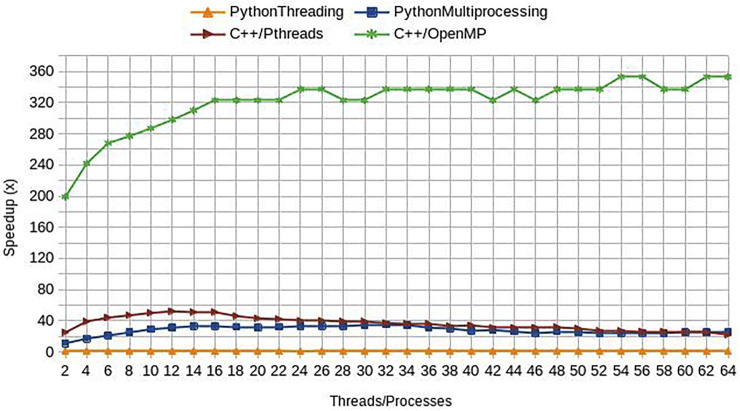
Performance of new Geomfinder implementations. The original (PythonThreading) and three new versions, including parallel programming paradigm and code optimization, were compared against a sequential Python version (Sequential) developed with benchmarking purposes. These versions were built using Python 2.7 with multiprocessing (PythonMultiprocessing), C++ with POSIX threads (C++/Pthreads), and C++ with OpenMP annotations (C++/OpenMP). As is denoted by the green line in the graph, the version C++/OpenMP always showed better performance than the other implementations, reaching their best acceleration using 54 threads: 353x speedup compared to Sequential version. Thus, the version C++/OpenMP was selected for the implementation of the new server of Geomfinder. The experimental setup used in the comparisons is a 32 Intel Xeon CPU E5-2683 (2.10 GHz) SMP system with hyperthreading enabled (64 virtual cores/threads), 252 GB RAM, 40 MB Intel Smart Cache.

All the new versions of Geomfinder, implemented with benchmarking purposes, incorporated several optimizations such as more efficient data structures and compilation flags for the machines utilized on the webserver. As denoted by the green line in Figure 4, the version C++/OpenMP always showed a better performance than the other implementations, achieving a maximal 353x speedup compared to the original Sequential version (Supplementary Figure S6). On this basis, the C++/OpenMP version was selected for the implementation of the new server of Geomfinder.

### Case of Use

**FIGURE 5:**
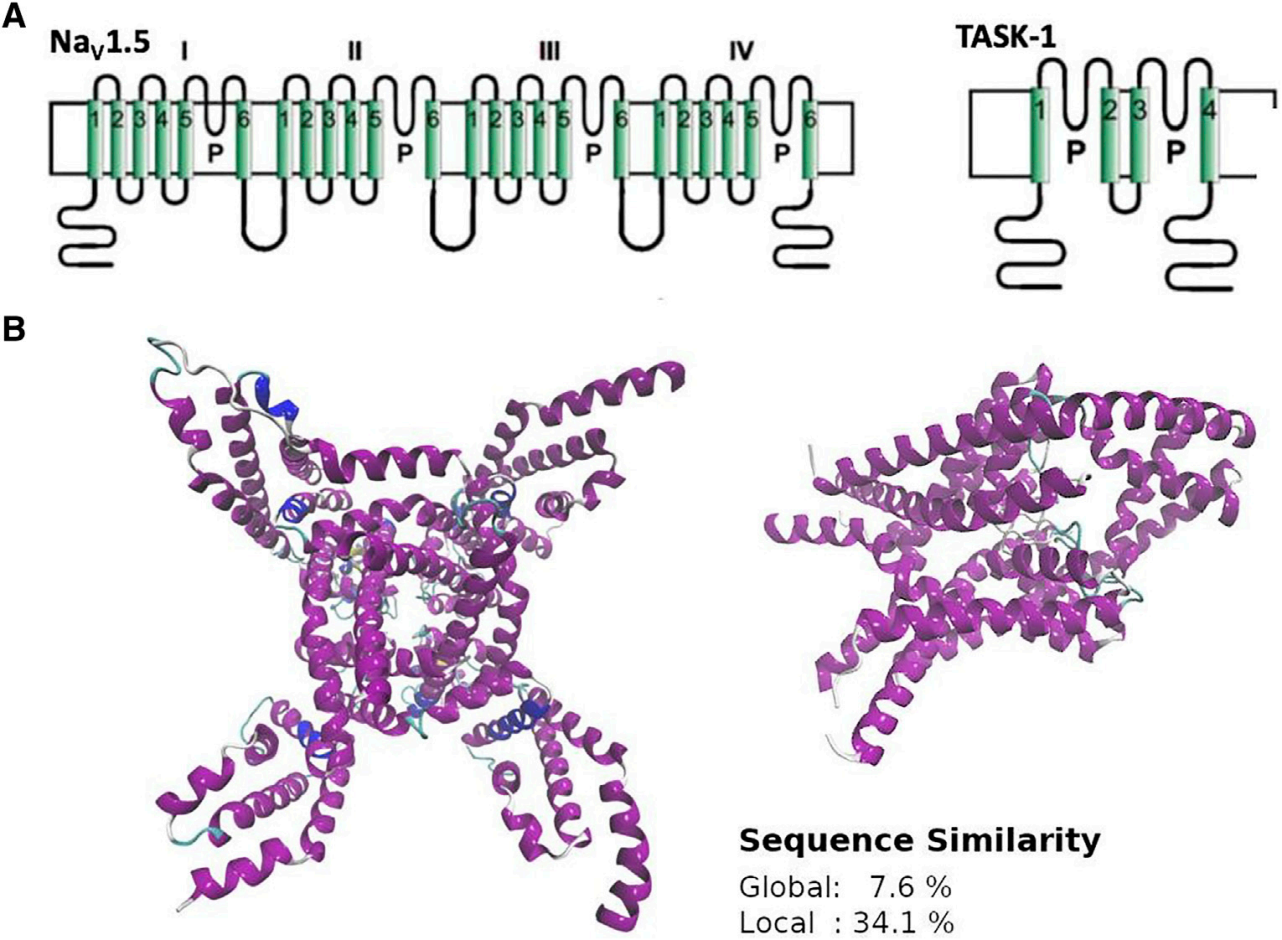
General overview of structure **(A)**, topology **(B)** and sequence (inset) similarity of NaV1.5 and TASK-1. NaV1.5 corresponds to the PDBid:6UZ3 and TASK-1 to the PDBid:6RV3. NaV1.5 is a monomer with four domains, each containing a pore sequence and TASK-1 is a dimer with four transmembrane segments and two pore sequences each monomer.

It has been shown that some local anesthetics are multi-channel blocking drugs, which interact with cardiac ion channels such as NaV1.5 and TASK-1 (Tikhonov and Zhorov, 2017; Rinné et al., 2019). Considering that this polypharmacological profile likely underlies their antiarrhythmic effect, we searched similar and druggable 3D patterns between these channels. Although NaV1.5 and TASK-1 have different sequences, structures, and topologies (Figure 5), Geomfinder required a few seconds to detect several similar 3D patterns (Supplementary Figure S2).

**FIGURE 6:**
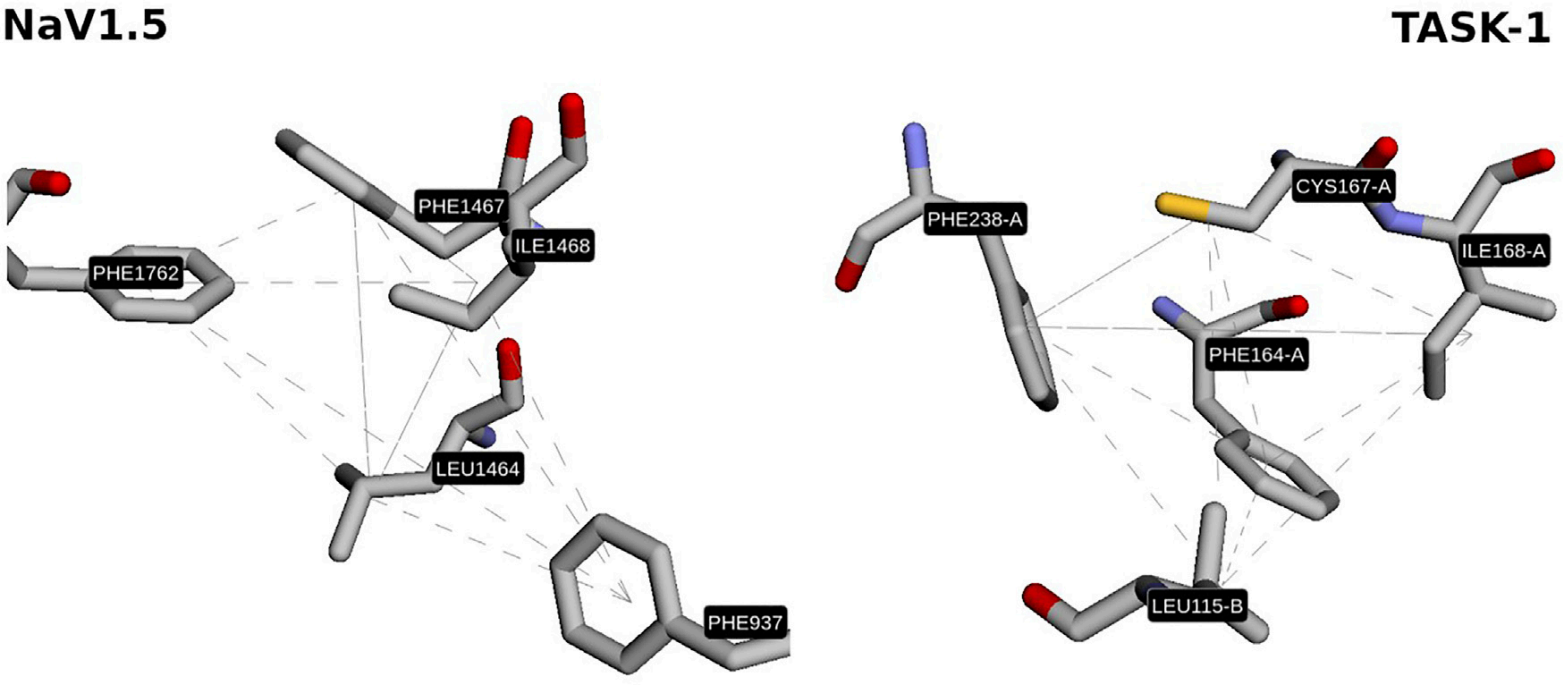
Similar 3D pattern found in NaV1.5 and TASK-1. In both sites, the residues PHE1762 and PHE238 show similar orientation.

Interestingly, one of the patterns found contains two key residues of the local anesthetics binding site in NaV1.5 (Phe1762, Ile1468; (Nguyen et al., 2019)) and one key residue in TASK-1 (Phe238; (Rinné et al., 2019)) located at the fenestrations. Considering that these 3D patterns are similar (GScore = 66.5%) and are located in zones with high druggability values (Phe1762 and Ile1468 are located in pockets with 99.9% druggability in NaV1.5; and Phe238 in a pocket with 100% druggability in TASK-1; result #25 in Supplementary Figure S3), it is enticing to state that the use of these 3D patterns is a promising starting point to understand the polypharmacological profile of local anesthetics and to design/search new multitarget compounds aimed to these cardiac ion channels. The fact that the residues Phe1762 in NaV1.5 and Phe238 in TASK- 1 were found by Geomfinder in similar tridimensional orientations into each 3D pattern (Figure 6), supports the idea that the aromatic ring of local anesthetics interacts with the ion channels establishing a π−π interaction (González et al., 2001).

**FIGURE 7:**
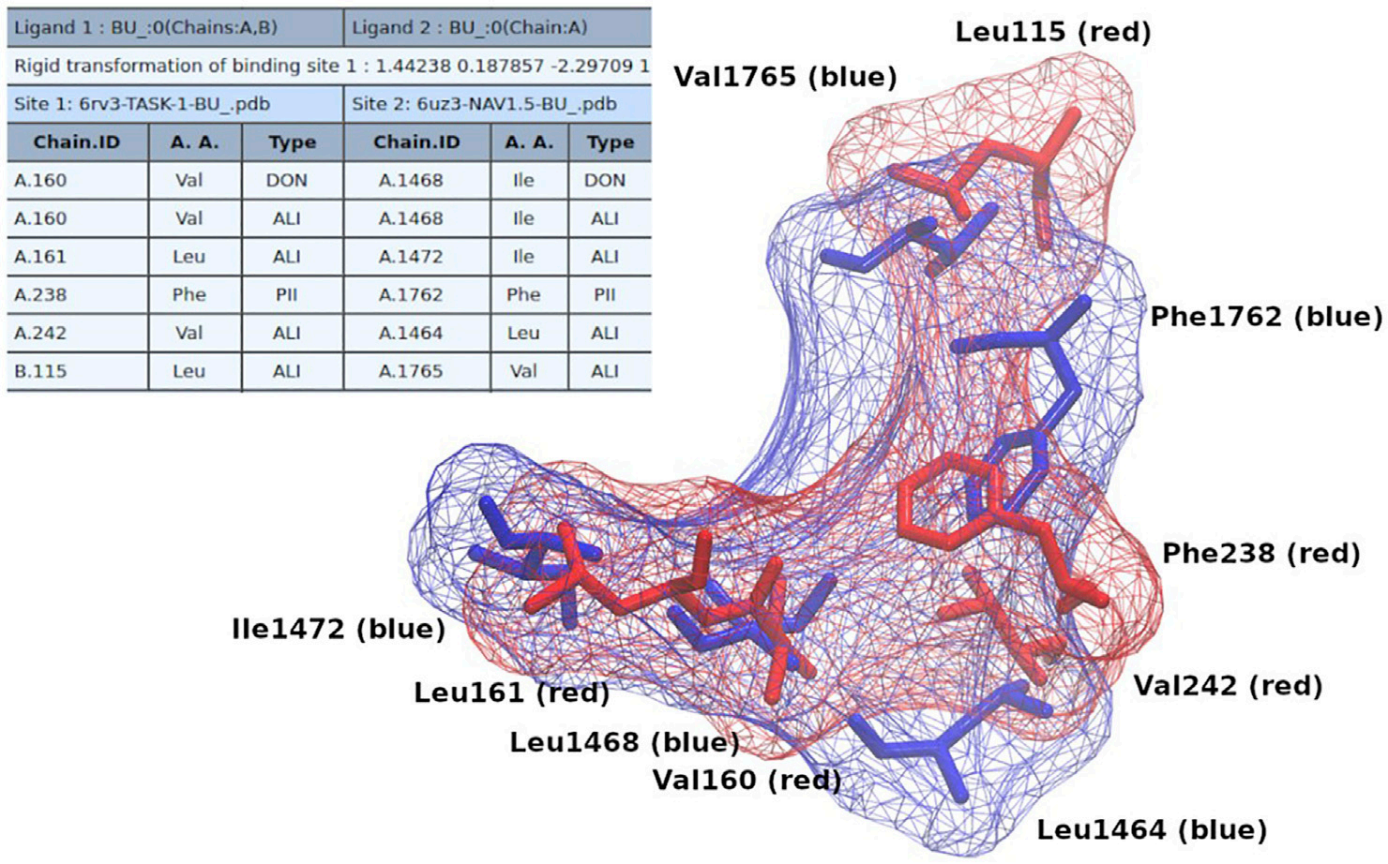
Common binding site of bupivacaine on TASK-1 and NaV1.5. The table (from Multibind) indicates the equivalent physicochemical properties matched. The colored residues depicted in licorice show the alignment (from PocketAlign) of the bupivacaine binding sites on both proteins (Red: NaV1.5; Blue: TASK-1).

In order to show how these results could be useful for the design/search of new multitarget compounds, we initially performed docking molecular simulations of bupivacaine (a common local anesthetic) on NaV1.5 and TASK-1 using the coordinates of the similar pockets detected with Geomfinder as grid centers. As shown in Supplementary Figures S4, S5, the aromatic ring of the bupivacaine seems to be establishing π-π interactions with Phe238 in TASK-1 and with Phe1762 in NaV1.5. After selecting the best conformers of bupivacaine (those with the lowest estimation of free energy of binding) in both proteins, we extracted the residues located at 4 Å of the ligands, and constructed a common binding site for TASK-1 and NaV1.5, as we have previously described (Möller-Acuña et al., 2015; Núñez-Vivanco et al., 2018). Briefly, this common binding site was constructed using external computational tools such as PocketAlign (Yeturu and Chandra, 2011) and MultiBind (Shulman-Peleg et al., 2008), which perform several structural alignments and tridimensional rearrangements, looking for the best match of the physicochemical properties of the selected binding sites. As shown in Figure 7, it was possible to find a tridimensional fit for adjusting the physicochemical properties and the structural alignment of the residues of both binding sites, which we call the common “receptophore” (Núñez-Vivanco et al., 2018). After that, we used this “receptophore” to perform a structure-based virtual screening with the e-LEA3D tool (Douguet, 2010). This software requires as input a PDB file of one pocket structure, which can then be used to make either a *de novo* drug design or a virtual screening into a collection of known compounds, which in both cases should lead to find molecules with high potential affinity for the pocket. In our case, we merged both aligned binding sites into one unique PDB file and after run e-LEA3D in the virtual screening mode (using the “FDA approved drugs” data set available at the e-LEA3D web server), a list of molecules ranked by their theoretical affinities for the pocket submitted was obtained. The top ranked molecule was tafluprost (Drugbank_ID: DB08819), a prostaglandin F_2α_ (PGF_2α_)-type agonist currently used as a treatment for glaucoma and ocular hypertension (Papadia et al., 2011; Klimko and Sharif, 2019), which in theory should be, a new putative multitarget ligand of NaV1.5 and TASK-1.

**FIGURE 8:**
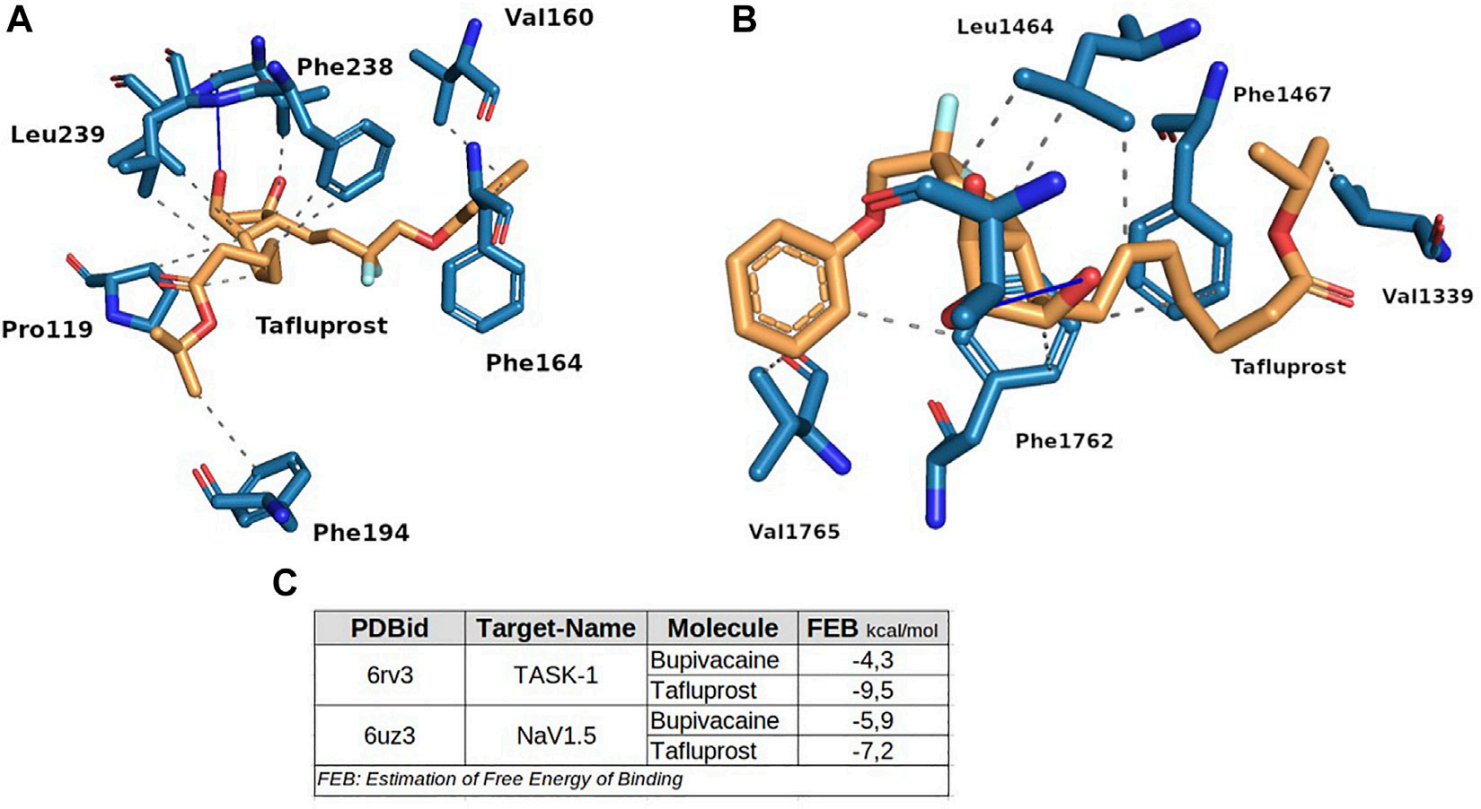
Binding modes of tafluprost on TASK-1 **(A)** and NaV1.5 **(B)**. The box at **(C)** shows the estimation of Free Energy of Binding of bupivacaine and tafluprost for both proteins. These experiments were performed at the same conditions.

To test this idea, we performed new docking molecular simulations with tafluprost on the structures of NaV1.5 and TASK-1, setting the same parameters that were used for the experiments with bupivacaine. As stated in Figure 8, tafluprost showed better affinities than those obtained for bupivacaine at both protein structures. The stabilization of the protein-ligand complexes seems to be determined by several hydrophobic interactions of residues which have been previously reported as key residues for the local anesthetics (e.g. Phe238, Leu1464 and Phe1762).

### Experimental Evaluation of Tafluprost

**FIGURE 9:**
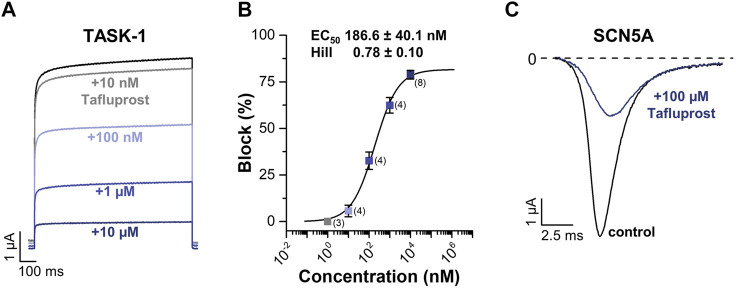
**(A)** Representative current traces of TASK-1 in the absence (black) or the presence of increasing concentrations of tafluprost (colored). **(B)** Percentage of block of outward currents in TASK-1 channel by tafluprost. Each point is the mean ± SD of 3-8 determinations. **(C)** Representative current traces of NaV1.5 (also referred as to SCN5A) before (black) and after (blue) application of 100 µM tafluprost.

As shown in Figure 9, tafluprost potently blocked TASK-1 outward currents in a concentration-dependent manner (EC_50_ = 186 ± 40 nM; Figures 9A,B). It should be noted that since the highest tafluprost concentration used in these experiments was 10 µM (Figure 9B), here we report EC50 instead of IC50, as the hill fit determines the concentration where tafluprost reaches 50% of its maximal effect but not 50% inhibition. As we reached 82% maximal inhibition, EC50 concentration corresponds to about 40% inhibition (not 50% inhibition). On the other hand, tafluprost also blocked NaV1.5 inward currents, although with much less potency than that observed at TASK-1 (estimated IC50 of about 76 µM Figure 9B).

## Discussion

The search of multitarget compounds might be a difficult task, particularly when the drugs are aimed to act at receptors with highly diverse structure and function. Based on the idea that a given compound could simultaneously interact with two (or more) relevant targets if they have similar binding sites (Jalencas and Mestres, 2013; Salentin et al., 2014; Ehrt et al., 2016; Konc, 2019; Naderi et al., 2019), one reasonable approach to find promiscuous drugs under these circumstances is to look for similar binding sites at the addressed targets. In this context, Geomfinder2.0 appears as a valuable tool since it is a fast web server for the discovery of similar and druggable 3D patterns between any pair of protein structures. This new version has significantly improved its usefulness and performance as compared with the original version, with up to 353x speedup for the input data set analyzed and the available machine, and allowing to compare a list of pairs of structures. It also identifies 3D patterns formed by different protein chains and characterizes how druggable is the zone where the 3D patterns were detected. It is important to note that, beyond these functional and performance improvements, the core method for searching and comparison of 3D patterns is the same as in the original version (Núñez-Vivanco et al., 2016). The accuracy and precision of this core method has already been compared with those of computational tools such as PocketMatch and ClickTopology (Núñez-Vivanco et al., 2016). In addition, in the present work we confirmed the reliability of our algorithm with a case of use, in which the theoretical predictions were experimentally confirmed.

Interestingly, when analyzing TASK-1 and NaV1.5, two cardiac channels with highly different structures and functions, Geomfinder2.0 was able to find (in a few seconds) several similar 3D patterns, a pair of which seemed remarkably attractive since they included some key residues involved in the binding of local anesthetics at both types of ion channels (Nguyen et al., 2019; Rinné et al., 2019). On this basis, we dissected these 3D patterns and constructed a common binding site which was used to search for possible novel multitarget ligands. As the development of novel polypharmacological agents can be a difficult, time-consuming and expensive task, drug repurposing (i.e. the establishment of new indications of existing drugs), has been proposed as an efficient alternative over the *de novo* drug development approach. Thus, using a “FDA-approved” data set we searched for known compounds that might show an unanticipated ability to interact with TASK-1 and NaV1.5. Tafluprost, a prostaglandin analogue (PGF_2α_ agonist) currently used for the treatment of open-angle glaucoma (Papadia et al., 2011; Klimko and Sharif, 2019), was the most promising drug arising from this analysis. Remarkably, when experimentally tested tafluprost blocked the corresponding currents at both NaV1.5 and TASK-1, although with a higher potency for the latter. The differential activity of tafluprost upon both ion channels (as well as its higher potency as compared with the local anesthetic bupivacaine (Stoetzer et al., 2016)) roughly agrees with the theoretical binding energies predicted by the docking simulations. Noteworthy, although tafluprost was clearly less active in NaV1.5 than in TASK-1, it still shows a potency in a similar range as that shown by the well-known sodium channel blocker bupivacaine (Zhang et al., 2014). Even though this is the first time that an activity of tafluprost on cardiac channels is described, it should be noted that it had been reported that the drug is able to induce a relaxation of rabbit ciliary arteries precontracted with a high-potassium solution (Dong et al., 2008). Accordingly, this effect might be related with its potent TASK-1 blocking properties.

Both NaV1.5 and TASK-1 are attractive drug targets in particular for the development of treatments of atrial fibrillation, the most common cardiac arrhythmia (Sossalla et al., 2010; Wiedmann et al., 2021). In addition, multichannel blockers such as amiodarone or dronedarone have been used in clinical settings (Kraft et al., 2021). In this context, the pharmacological activity predicted and demonstrated here for tafluprost, suggests that this compound could be a good candidate to evaluate its properties and repurpose it as a novel antiarrhythmic drug.

In summary, despite mounting evidence indicating that polypharmacological drugs might show better efficacy and less side effects than more selective compounds, early work in this area had already recognized that the rational search of multitarget drugs faces at least two major challenges, including a) the need to identify a combination of nodes in a biological network whose perturbation results in a desired therapeutic outcome, and b) to develop drugs whose polypharmacological profile allows those nodes to be perturbed specifically (Hopkins, 2008). Therefore, computational tools such as Geomfinder can be helpful to discover similar and druggable 3D patterns among proteins which have been tagged as targets in a multi-factorial disease, which appears as an important first step to either rationally design or -as in this case-purpose novel indications for compounds already in use.

## Data Availability

The datasets presented in this study can be found in online repositories. The names of the repository/repositories and accession number(s) can be found in the article/Supplementary Material.
