# Supplementary material for: A New Strategy for Multitarget Drug Discovery/Repositioning Through the Identification of Similar 3D Amino Acid Patterns Among Proteins Structures: The Case of Tafluprost and its Effects on Cardiac Ion Channels
Source: Front Pharmacol. 2022 Mar 18;13:855792. doi: 10.3389/fphar.2022.855792 (PMC8971525; doi:10.3389/fphar.2022.855792)
Supplement: Supplementary file 1 [file DataSheet1.docx]

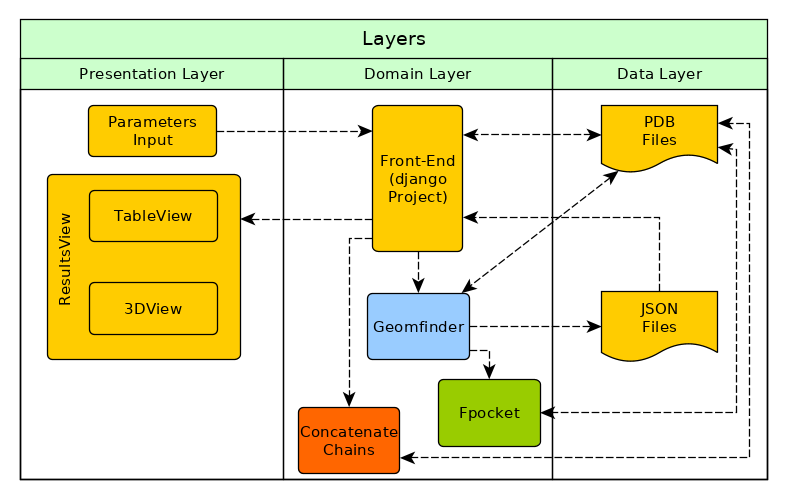


**Supplementary Figure S1.** This representation is divided into three main layers: the presentation, the domain, and the data layer: *The presentation layer* represents the user’s view and the interaction with the *domain layer*. It consists of two modules: *ParametersInput*: is responsible for obtaining the necessary data to compute the request. *ResultsView*: is composed of the following items: *TableView*: data tables used to show the list of jobs executed and the 3d patterns found. *3DView*: a tool for the 3D structural visualization*. The domain layer* represents the core of Geomfinder2.0 and denotes the communication link between the *presentation* and *data layers*. This layer has the following components: *Front-End*: This module is the web platform developed using the framework Django. It allows users to upload, process and visualize the input data and the results. *ConcatenateChains*: This module allows concatenating a set of user-defined chains of a protein. *Fpocket*: This module identifies all possible cavities (pockets) on the structure of proteins. Each cavity has a score named “Druggability Score” which is used to filter the results. *Geomfinder*: This module is the main process and receives the output of the *ConcatenateChains* module. Also, it can use, if the user selects this option, the *Fpocket* module results**.** The *data layer* has two components which are responsible for store the data: *PDB files*: This type of files is used both as the input (proteins to compare) and output (pockets generated by fpocket program). *JSON files*: Format of the files that contain the processed results (mainly used on data tables).


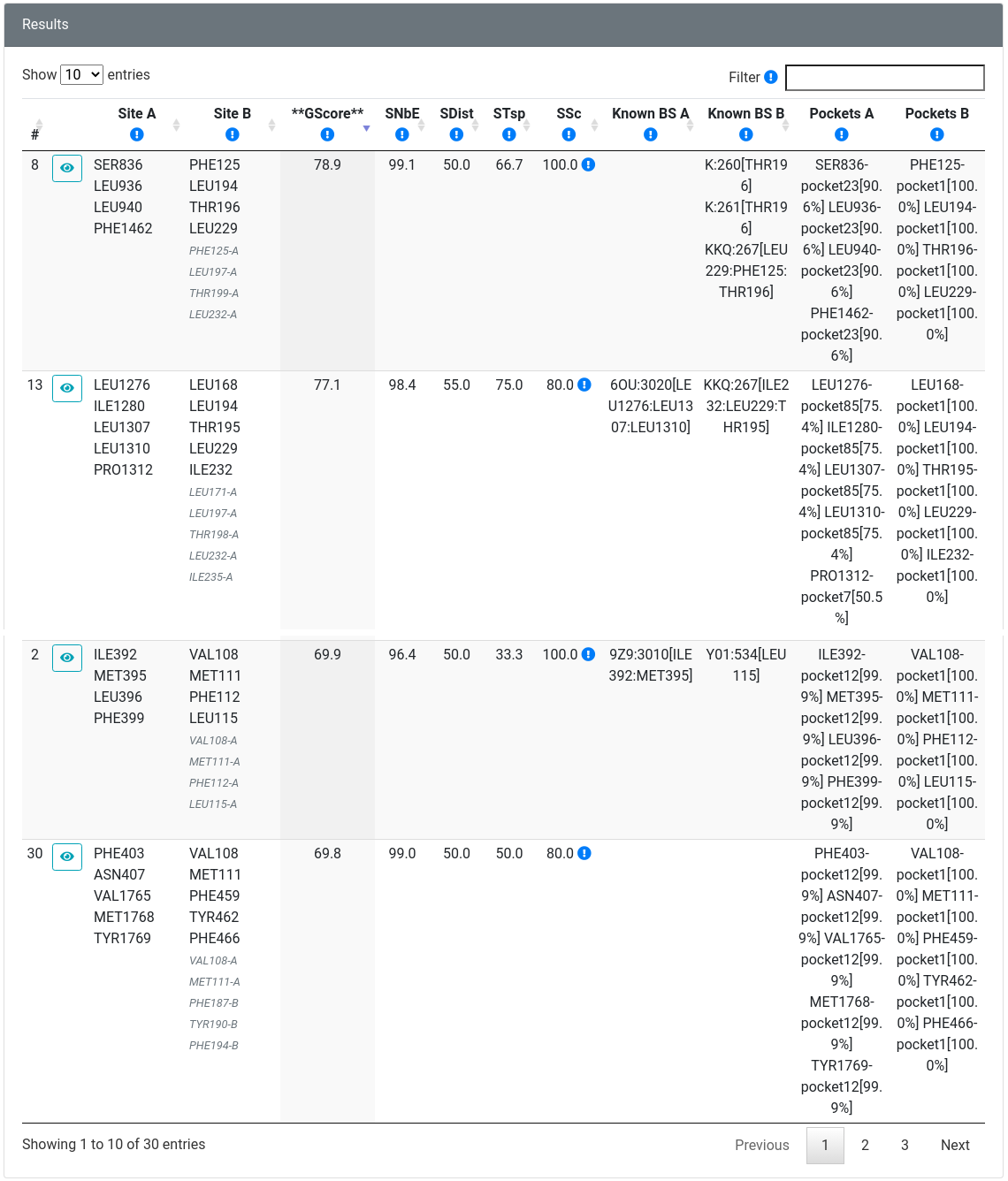


**Supplementary Figure S2.** This table shows the main result page of Geomfinder, where each pair of 3D patterns compared is listed. The list of pairs of 3D patterns can be filtered and sorted by different features (Amino acid components, GScore value, etc.).


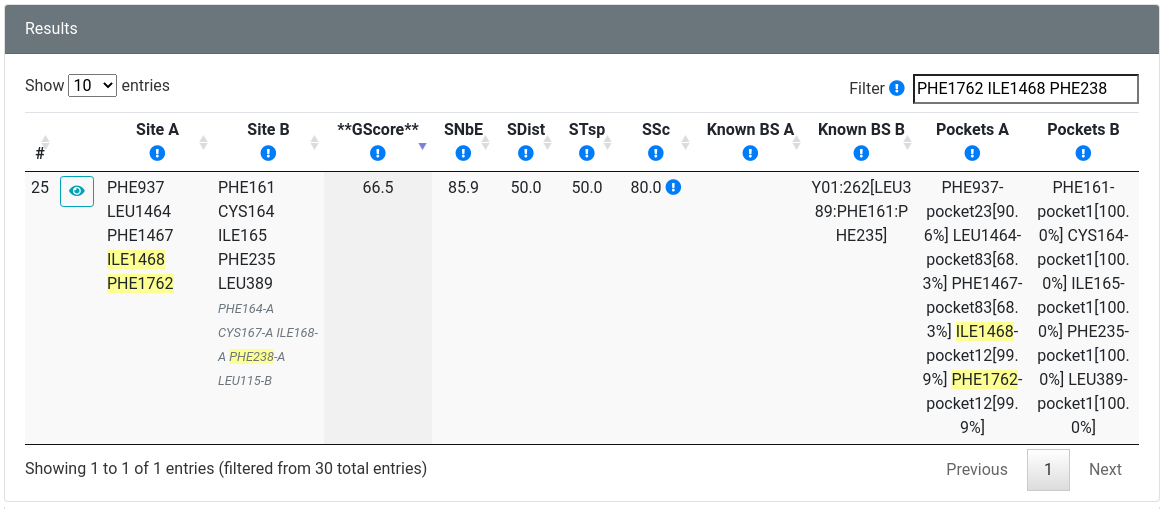


**Supplementary Figure S3.** The first result delivered by Geomfinder is a list of the 3D patterns found. As is shown in Figure S2, each pair of 3D patterns have a unique ID, the list of residues forming each site, the GScore, the particular scores of similarity (SNbE, SDist, STsp, SSc), the known ligands near of the site and the residues forming the cavities whose druggability scores are higher than those defined by the user. We selected the result #25 because the sites contain two critical residues of the local anesthetics binding site in NaV1.5 (F1762, I1468; Figure S3.2) and one essential residue in TASK-1 (F238; Figure S3.3) located at the fenestrations. The use of these 3D patterns is a promising starting point to understand the polypharmacological profile of local anesthetics in cardiac ion channels. Besides, as shown in Figure S3.1, F1762 in NaV1.5 and F238 in TASK-1 were found by Geomfinder in similar tridimensional orientations into each 3D pattern, which enhances the idea that the aromatic ring of local anesthetics, interacts with the ion channels establishing a Π – Π interaction.


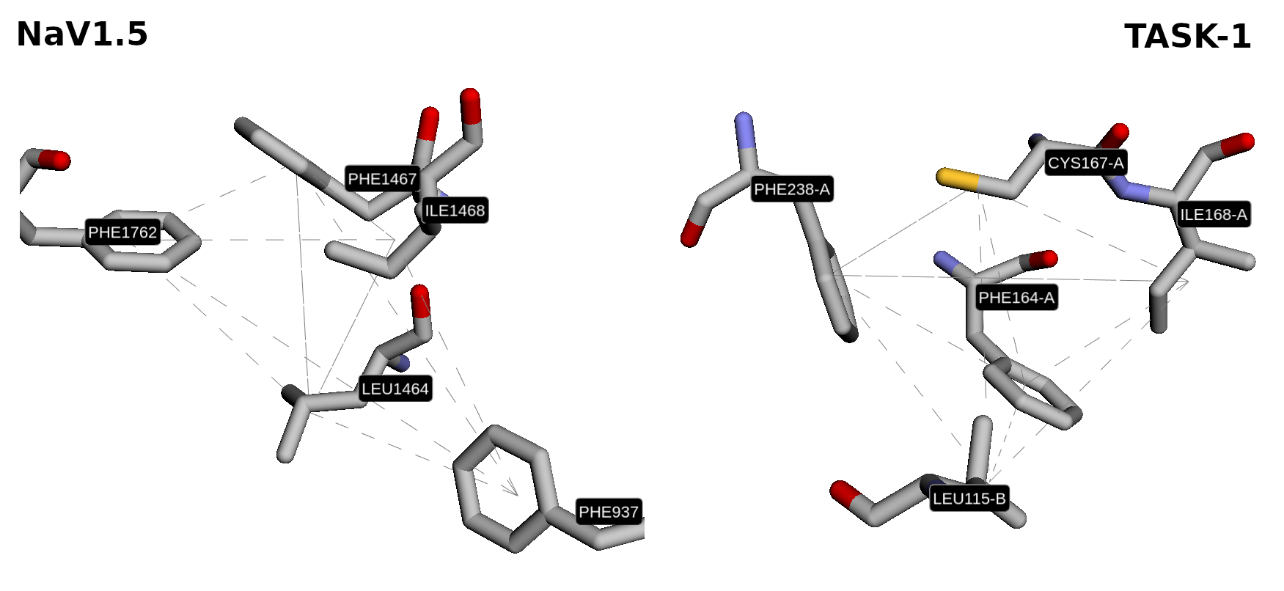


**Supplementary Figure S3.1.** Residues of the site A (Left, NaV1.5): PHE937 LEU1464 PHE1467 ILE1468 PHE1762. Residues of the site B (Right, TASK-1): PHE161 CYS164 ILE165 PHE235 LEU389 (Original number/chain: PHE164-A CYS167-A ILE168-A PHE238-A LEU115-B)


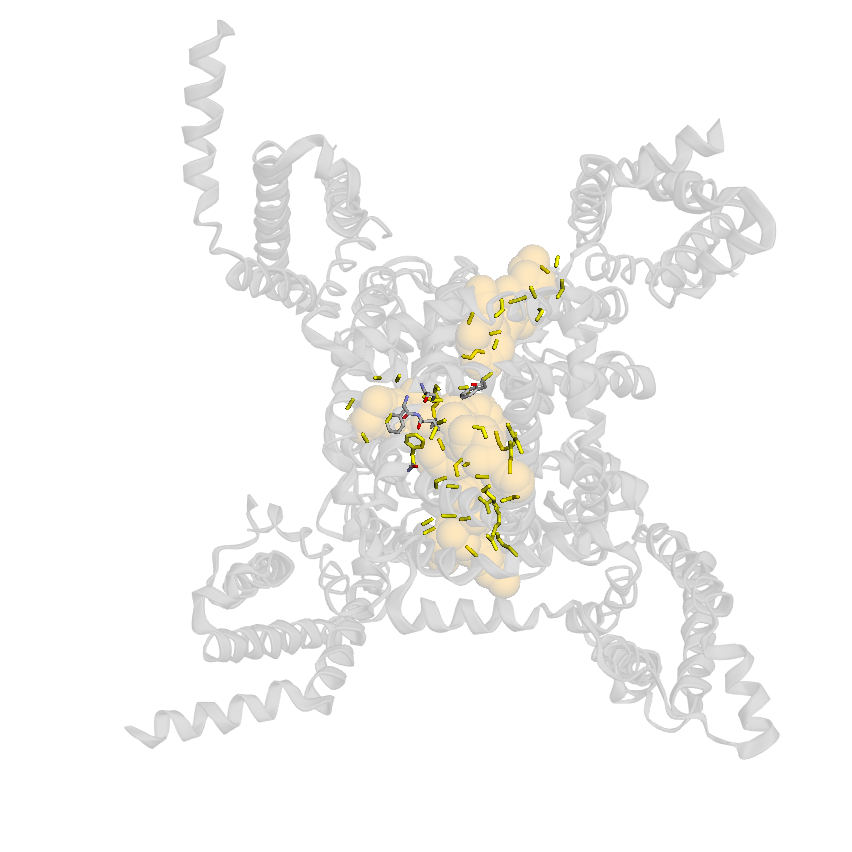


**Supplementary Figure S3.2.** Structure of NaV1.5 highlighting the anesthetics binding site and the 3D pattern where residues F1762 and I1468 were found.


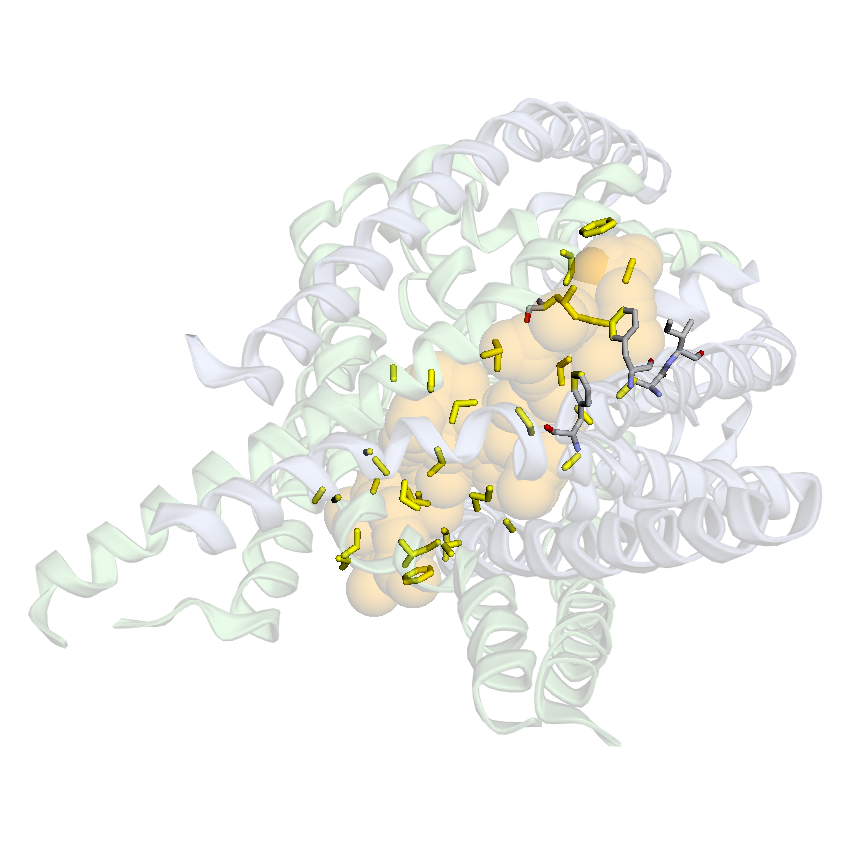


**Supplementary Figure S3.3.** Structure of TASK-1 highlighting the fenestration zone and the 3D pattern where residue F238 was found.


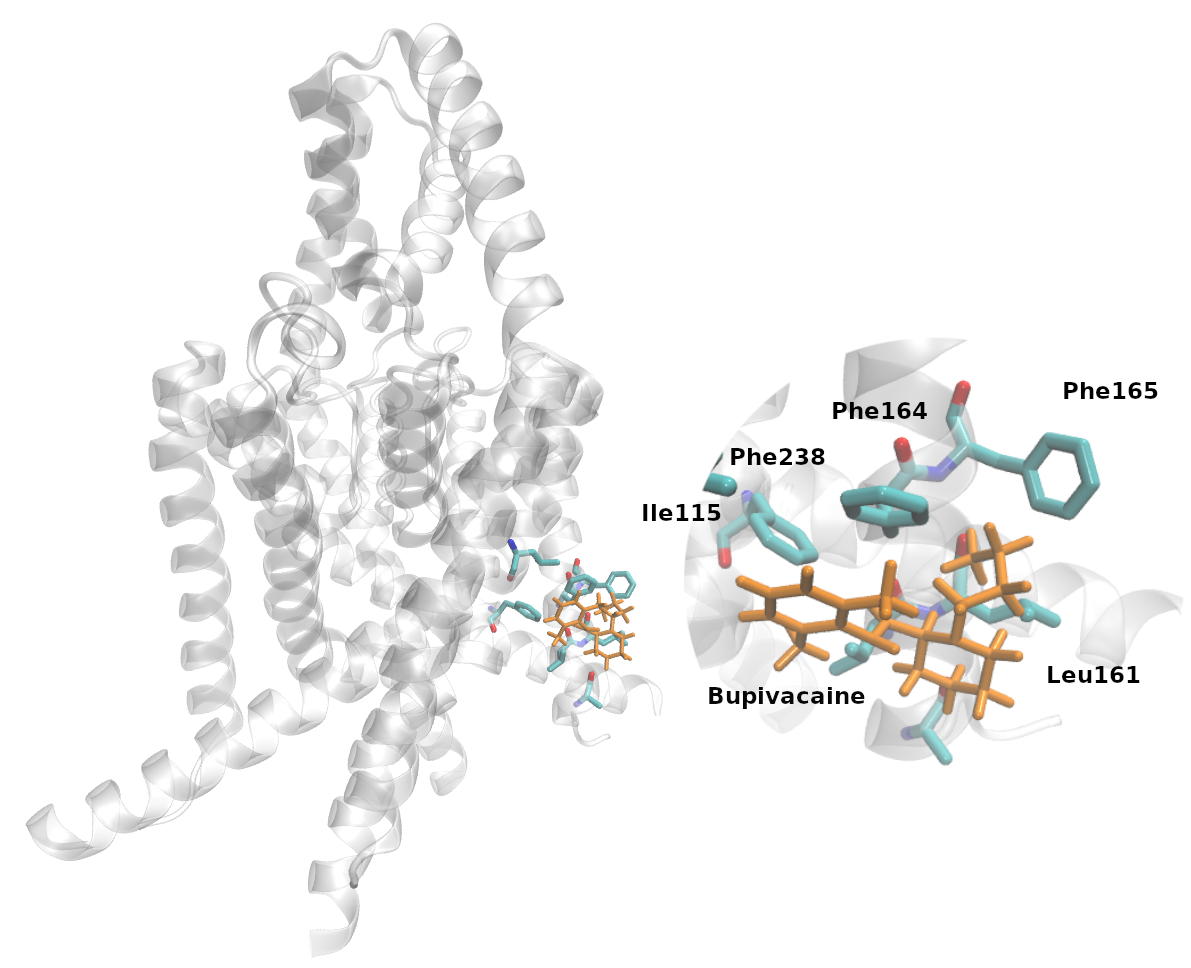


**Supplementary Figure S4.** Binding mode of Bupivacaine on TASK-1.


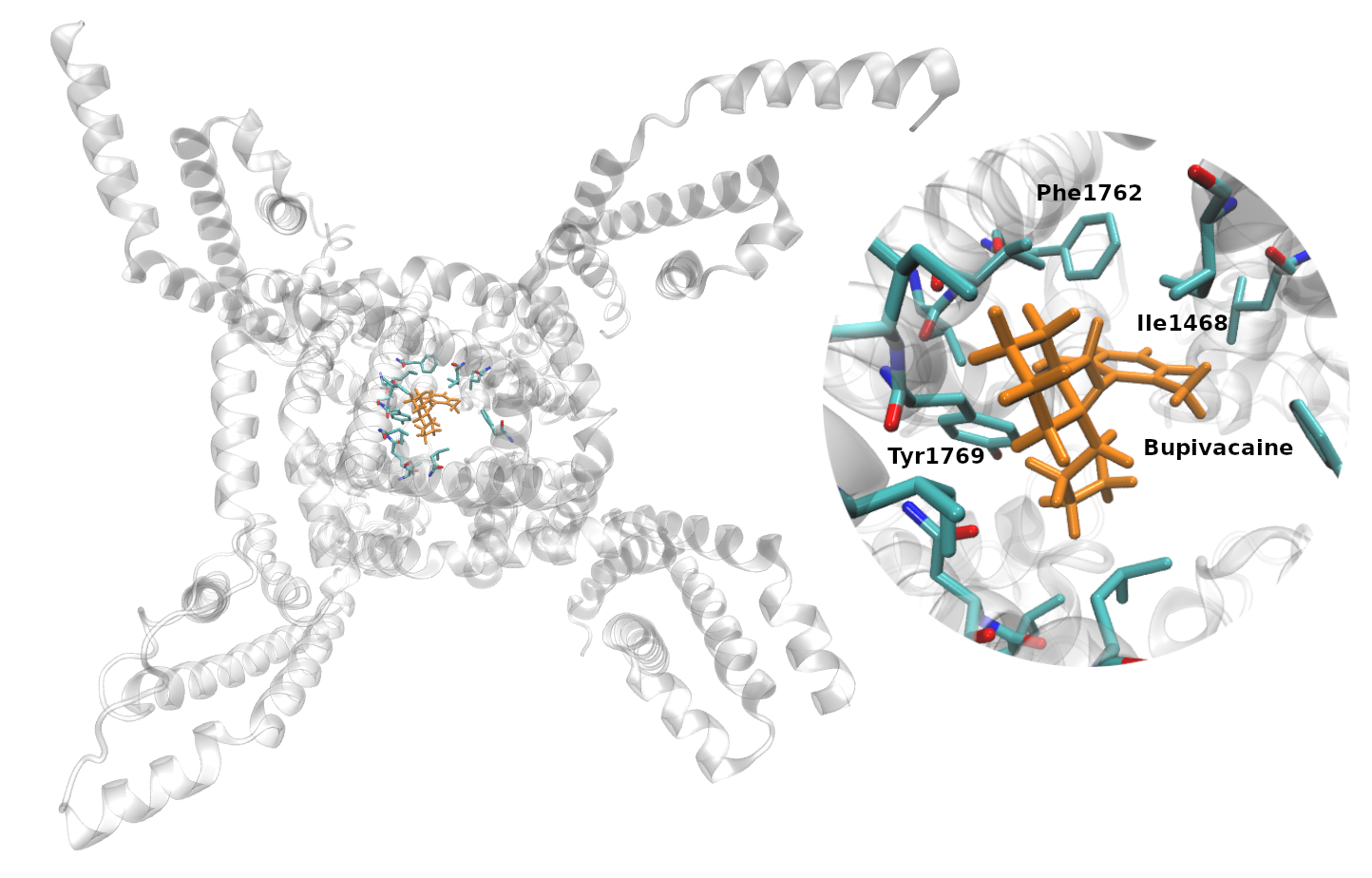


**Supplementary Figure S5.** Binding mode of Bupivacaine on NaV1.5.

**
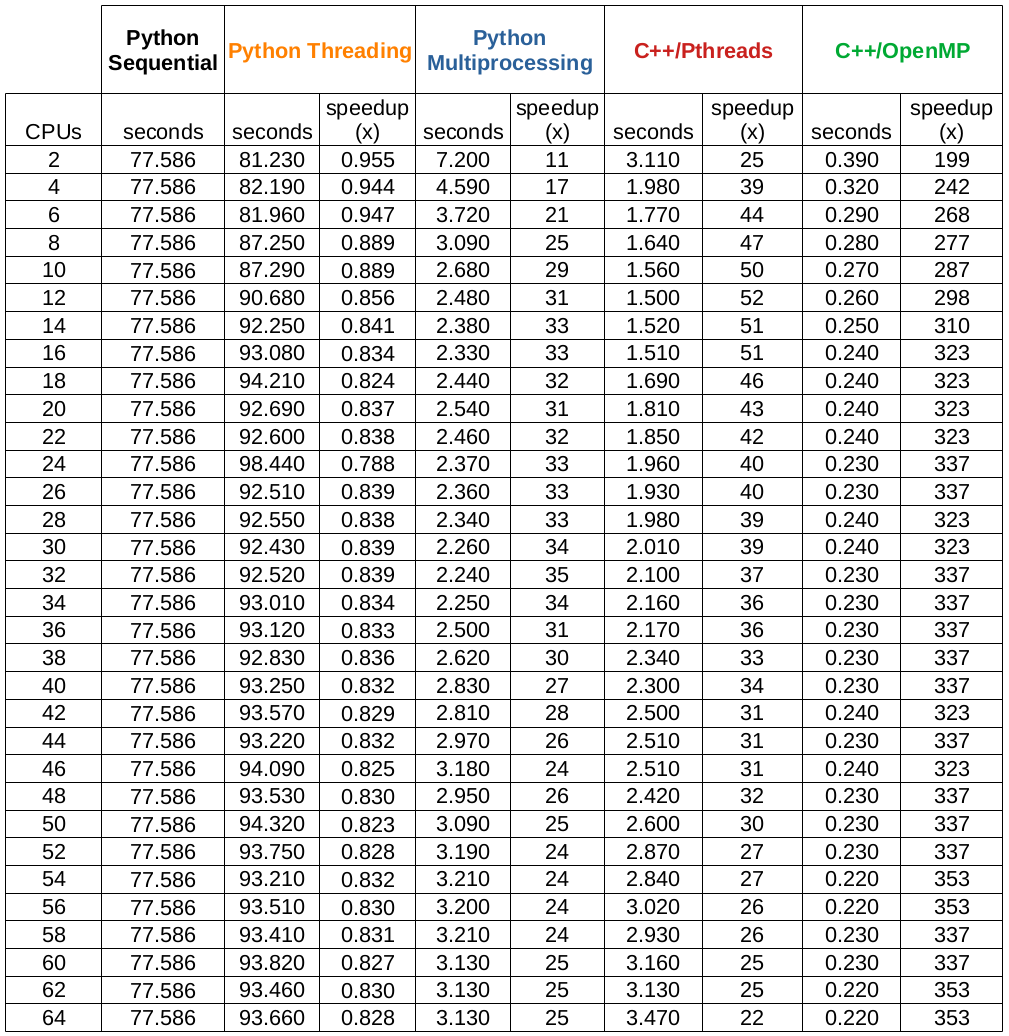
**

**Supplementary Figure S6.** Data table with the results of the benchmarks. Versions PythonThreading, PythonMultiprocessing, C++/Pthreads, and C++/OpenMP were compared to the PythonSequential version. Each row shows the elapsed time (seconds) and the speedup obtained using different amounts of CPU.
